# Resilient help to switch and overlap hierarchical subsystems in a small human group

**DOI:** 10.1038/srep23911

**Published:** 2016-04-05

**Authors:** K. Fujii, K. Yokoyama, T. Koyama, A. Rikukawa, H. Yamada, Y. Yamamoto

**Affiliations:** 1 Research Center of Health Physical Fitness and Sports, Nagoya University, Furo-cho, Chikusa-ku, Nagoya, 464-0814, Japan; 2 Research Fellow of the Japan Society for the Promotion of Science, Japan; 3 Sport Medical Science Research Institute, Tokai University, 4-1-1 Kitakaname Hiratsuka Kanagawa, 259-1292, Japan; 4 School of Physical Education, Tokai University, 4-1-1 Kitakaname Hiratsuka Kanagawa, 259-1292, Japan

## Abstract

Groups of social organisms in nature are resilient systems that can overcome unpredicted threats by helping its members. These social organisms are assumed to behave both autonomously and cooperatively as individuals, the helper, the helped and other part of a group depending on the context such as emergencies. However, the structure and function of these resilient actions, such as how helpers help colleagues and how the helper’s action is effective at multiple subsystem scales remain unclear. Here we investigated the behaviour of organised and efficient small human groups in a ballgame defence, and identified three principles of hierarchical resilient help when under attack. First, at a present high emergency level, the helper simply switched the local roles in the attacked subsystem with the helped. Second, at an intermediate emergency level, the helpers effectively acted in overlapping subsystems. Third, for the most critical emergency, the helpers globally switched the action on the overall system. These resilient actions to the benefit of the system were assumed to be observed in only humans, which help colleagues at flexibly switched and overlapped hierarchical subsystem. We suggest that these multi-layered helping behaviours can help to understand resilient cooperation in social organisms and human groups.

Groups of living organisms can achieve a greater quality of work than individuals. This ability has been called collective intelligence^1^, defined as the general ability of a group to perform a wide variety of tasks in mobile animals^2^,^3^,^4^,^5^, robotics^6^,^7^,^8^,^9^, simulated agents^10^,^11^ and human groups^1^,^12^,^13^,^14^,^15^,^16^ operating as autonomous decentralised systems. For example, interactions between homogeneous mobile animals can solve problems in complex environments^2^,^3^ by entrainment with the movements of neighbours. However, the individuals who make up groups in nature are sometimes considerably heterogeneous because of role-sharing, e.g. competition^17^,^18^, cooperation^19^ and playing various specific roles^9^,^14^,^15^,^16^,^20^,^21^,^22^. Even if a primary goal is simple, such as chase or escape^21^,^23^, autonomous role-shared agents adapt to the behaviours of the enemy and colleague, and to other changing factors such as heterogeneous environment^24^,^25^. Consequently, each agent has multiple hierarchical lower-level goals^18^,^26^ in addition to the primary goal. Furthermore, in a well-organised system, agents should alternately or simultaneously behave as autonomous individuals, help or are helped in cooperative dyad and work dependently as part of the whole. These autonomous *and* dependent multi-agents in role-sharing^27^ to jointly act^28^ at appropriate subsystem scales (from the individual to the overall system) are necessary to solve problems based on hierarchical goal as an overall system; in nature, however, these joint actions remain unclear. The flexible system behaviour can be investigated by observing how role-shared agents in efficient social systems, such as organised small human group^14^, cope with various emergencies.

A resilient systems, such as an organism in nature^29^, network systems^30^,^31^ or adaptive robot^32^ can recover from unpredicted difficulties or impairment. Resilience has been studied as the capacity of a system to absorb disturbances and reorganise while undergoing change so as to maintain the same function, structure, identity and feedbacks^33^. Similarly, resilient multi-agent systems can overcome an emergency of intended or unpredicted attack, which is an uninvestigated type of collective intelligence. The important feature is how agents should cooperatively interact, because the social interaction may have sometimes negative effects^34^,^35^ on the whole system. In cooperative animal groups in nature, the resilience of the group is understood as helping behaviour such as observed in social wasp^20^ or brown hyenas^22^ despite being non-primate and/or with no kin relationship. The helping behaviour explicitly solves problems of the involved subgroup^19^ and implicitly preserves the function of the overall group^20^. In a state of emergency, however, helpers themselves are threatened with additional helping costs. Especially in efficient system with minimising the cost because of limited number of agents, such as navigation teams at unpredicted sea^14^, the agents must execute a suitable action with appropriate timing. Furthermore, in contrast to the predetermined role-sharing navigation teams requiring different expertise, we hypothesised that a helper’s behaviour in more flexible role-switchable system takes a variety of forms at appropriate subsystem scales. However, the structure and function of the resilient action, such as how helpers help colleagues, and how the helper’s action can be effective at multiple subsystem scales, remain unclear. Understanding these resilient helping in nature enables us to identify how groups of social organisms adapt to and survive (or manage the risk) inherent in confronting enemies or unpredicted crises.

In this study, we show the resilient helping behaviour in heterogeneous groups at multiple subsystem scales against intended attacks by observing sophisticated teams engaged in basketball game (Fig. 1a,b). Because of small limited number of members, sports teams provide excellent examples of how autonomous agents act in close and efficient coordination in a flexible role-switchable system with minimising personal costs. We focused on players performing person-to-person defence against an offensive team, where the roles of all defenders are predetermined to mark each attacker (i.e. individual role-filling). In a competition involving experienced teams, both defensive and offensive teams employ multi-layered strategies based on the primary goal of protecting all shootable spaces (i.e. cope with emergency in the attacker’s shot) and of creating a space for taking a shot, respectively. Thus, the defensive teams provide an example of showing the structure and the function of the resilient individual actions when confronting the attack of the offensive team. To investigate the overall defender’s behaviour, we first examined the degree of threat for a defensive team against competitor’s attacks using small competition networks^36^. We then analysed defensive subsystem coping behaviour with ‘screens’ as intended competitor’s subgroup attack, which is used to disrupt defensive players by blocking them.

## Results

### Degree of threat at multi-subsystem scales

**Figure 1:**
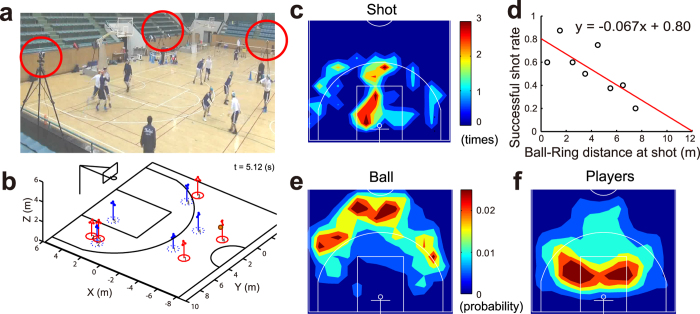
Measurement. (**a**) Experimental setup. Ten players and a ball were captured. Three red circles indicate optical motion cameras (6 cameras in total). Players wearing a white or blue sportswear performed half-court 5-on-5 basketball. (**b**) Coordinate system and example of optical motion capture system at the same time as (**a**). Players can be identified as the given numbers all of the times. (**c**) Heat map of shot position frequency. The shot was frequently taken near the 3-point line and near the ring. (**d**) Relationship between distance from ball to ring in shot and successful shot probability. The probability was linearly regressed (red line) with the distance. (**e**,**f**) Heat map of ball (**e**) and all players’ position (**f**) frequency in entire period of analysis. The ball was distributed distantly from the ring probably because defensive players move to shut the ball away from the ring, whereas the players were distributed near the ring.

First, we illustrated that the probability and opportunity of the primary threat was heterogeneously distributed across the field. The probability of the successful shot as primary defensive threat (i.e. defender’s failure) was decreased with the distance from the ring (Fig. 1d, *R*^2^ = 0.57, *F*_1,6_ = 7.96, *p* = 0.030, 52 shots in total) and the shot was frequently taken near the 3-point line (ball was moved: Fig. 1c,e) and near the ring (players moved: Fig. 1f) probably because of the profit and probability trade-off 37. In this study, we assumed that the probability of the primary threat was linearly decreased with the target (i.e. ring).

**Figure 2:**
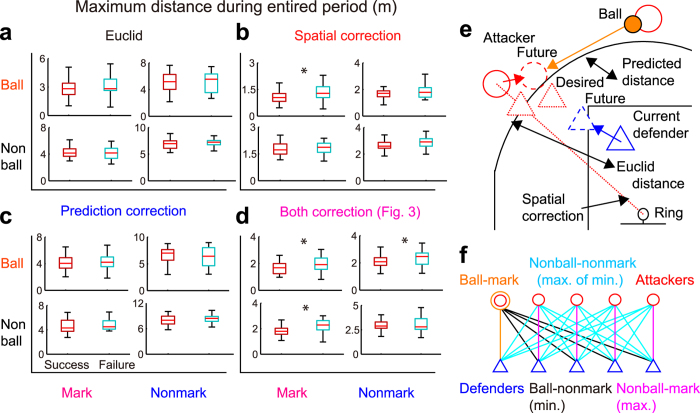
Maximal adjusted attacker-defender distance. (**a**–**d**) The 4 × 4 distance (corrections and subsystem scales) in successful (red) and failed (cyan) defences during entire period of analysis (for detail, see Supplementary Methods). Asterisk shows significant difference between the distance in successful and failed defences. Each distance diagram was shown in (**e**,**f**). (**a**) Euclid attacker-defender distance with no correction, (**b**) with spatial correction, (**c**) with prediction correction and (**d**) with both correction (Fig. 3) were shown. Results demonstrated that the maximum Euclid attacker-defender distances should be modified reflecting static-spatial specificity of shot probability and dynamic-predictive specificity of ball position. The detailed diagram of the distances at multi-subsystem scales were indicated in Fig. 3. (**e**) Diagram of the distance between current defender position (blue solid triangle) and desired defender position (red dot triangle) to prevent an attacker from shot and penetration into the ring is shown. The distance with four correction was calculated by the correction of spatial (red dot line: multiplying the successful shot probability in Fig. 1d) and/or prediction (reflecting ball, attacker and defender position and velocity). (**f**) Competition network diagram of the distance between attackers (circular node) and defender (triangle node) including the primary threat (ball) at 4 subsystem scales: ball-mark distance (orange), minimum ball-nonmark distance (black), maximum nonball-mark distance (magenta) and nonball-nonmark distance (blue) calculated by minimum (of non-mark) and maximum (of non-ball) distance.

Degrees of threat were explained as competitive inter-agent distance in heterogeneous field with static spatial- and dynamical predictive-specificity (Video S1). We examined various maximum attacker-defender distances during the entire period of analysis (for another interval, see Fig. S9) represented as the necessary spatial-gaps to avoid the primary threat. Results demonstrated that maximum Euclid attacker-defender distance should be modified reflecting static-spatial specificity of shot probability and dynamic-predictive specificity of ball position (Fig. 2 and Fig. S1 and Supplementary Text: *p* = 0.012, odds ratio = 6.38). In contextually homogeneous field, Euclid inter-agent distance is considered a critical parameter to control the qualitative change the dynamics of the whole system shown in mobile animal^2^,^3^ and human^38^,^39^ groups of homogeneous joint action^28^. However, our results indicated that in a physically and contextually heterogeneous field, the critical inter-agent distance to express the interaction should be adjusted considering static spatial-specificity and dynamical predictive-specificity.

**Figure 3:**
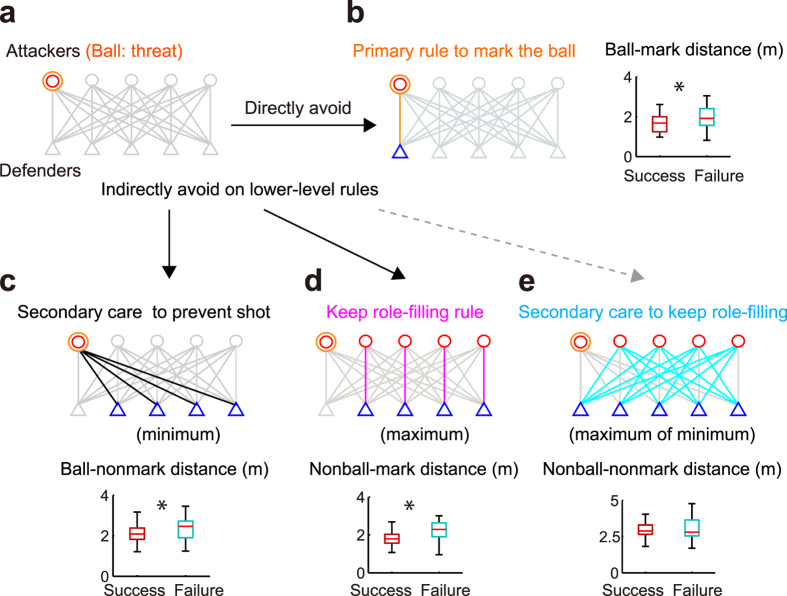
Attacker-defender distance. (**a**–**e**) Competition network diagram of the relationship between attackers (circular node) and defender (triangle node) including the primary threat (ball) at 4 subsystem scales: (**b**) ball-mark distance (orange); (**c**) minimum ball-nonmark distance (black); (**d**) maximum nonball-mark distance (magenta); (**e**) nonball-nonmark distance (blue) calculated by minimum (of non-mark) and maximum (of non-ball) distance. (**b**–**e**) The results of statistical analyses of adjusted distances between attackers and defenders at 4 subsystem scales in successful (red) and failed (cyan) defences were shown (for detail, see Supplementary Methods). Asterisks and black solid arrows show significant prediction between the distance in successful and failed defences.

Degrees of threat in competitive group were also expressed at multiple subsystem scales (Video S2) of competition network. Regarding the primary threat (Fig. 3a), the adjusted maximum distances at three different subsystem scales (Fig. 3b–d: ball-mark, ball-nonmark and nonball-mark) predicted significant difference between successful and failed defence (Table S2: all *p* < 0.29 and odds ratio > 2.98). The defender’s shortening of the adjusted ball-mark spatial-gap was directly effective to foil the shot, which was primary rule in defensive systems (Fig. 3b). In contrast, indirect coping with the threat on lower-level rules was observed (Fig. 3c,d: secondary care to prevent a shot, or keeping role-filling rule), which would be effective to avoid the system primary threat. Therefore, degrees of threat in competitive groups were expressed as the adjusted attacker-defender distance at multiple subsystem scales using small competition networks^36^. If defensive system has only one non-hierarchical rule, adaptive competitors will strike at a weak point (i.e. spatial-gap), such as the hub observed in the network research^40^. Thus, the system competing against adaptive competitors needs to select appropriate rules, which could create a more resilient system (e.g. switching the hub of a mobile group^41^). Below we demonstrate the resilience of the system against the specific emergencies of competitor’s subgroup attacks.

### Helping behaviour in a local emergency

Tactical competitor cooperation created hazardous emergency situations. Offensive teams used ‘screens’ to disrupt defensive players by legally blocking them (a total of 139 screens in 61 attacks) and to break defenders’ role-filling rules (Video S3). These screens represent an emergency for the defensive team. Before the use of the screen, the adjusted spatial-gaps of the involved agents did not differ between successful and failed defence (Fig. S2a); however, the adjusted ball-mark spatial-gap after the screen in failed defence was larger than those in successful defence (Fig. S2b and Table S2: *p* = 0.010, odds ratio = 5.34). Thus, specific tactically cooperative attack of the offensive team created defender’s direct primary threat (i.e. shot) to break the defensive organisation role-filling rules. Dysfunction of organisation would be lower-level emergency relative to the direct primary threat; however, our results showed that the dysfunction caused the hazardous crisis. Therefore, the system should find solutions at the involved subsystem scale to cope with the emergency, in other words, needs helping behaviours.

**Figure 4:**
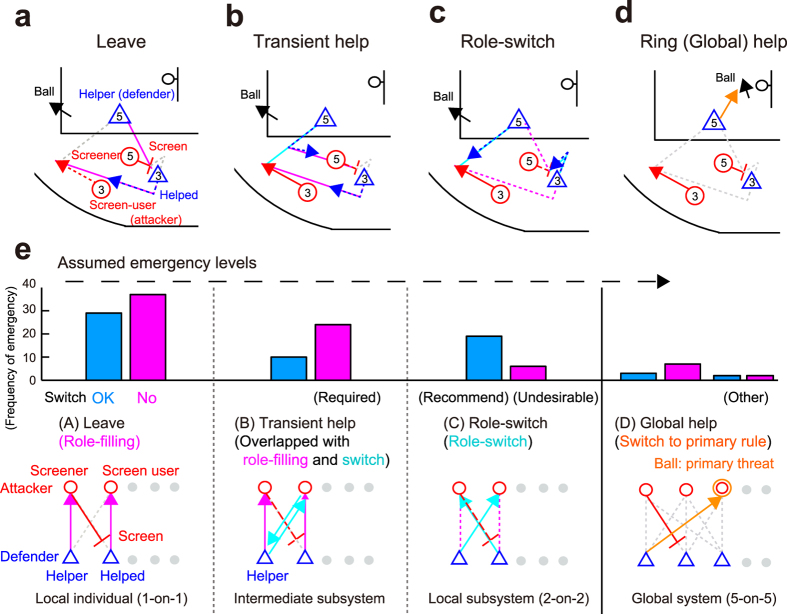
Patterns of helping behaviour with emergency. (**a**–**d**) Observed 4 cooperative patterns of coping dyad with the emergency: (**a**) leaving the screen, (**b**) transient help and return, (**c**) role-switching and (**d**) Global helping with the uninvolved colleague with the emergency (Other pattern is shown in Supplementary Materials). (**e**) Frequencies and competition subsystem network diagram of the 4 cooperative pattern organised at assumed emergency levels. The frequencies of transient help and role-switch were along with the favourable predetermined strategy (i.e. recommended role-switch was more than that in switch-avoided; in contrast, the relationship was reversed in transient help). The defensive team in this study predeterminedly gradated role-switching strategies not to be predicted by the competitors, i.e. switch-recommended (blue bar) or avoided (magenta bar).

Helping behaviour coping with the emergency showed various forms at multiple subsystem scales (Video S4,S5,S6,S7). We categorised four helping patterns coping with the competitor’s intended subsystem attack: (1) helper left the tactical attack (Fig. 4a, 66 screens, 47%); (2) helper transiently took the role of the helped and thereafter returned the original role (Fig. 4b, 34 screens, 24%); (3) helper completely switched roles with the helped (Fig. 4c, 25 screens, 15%); (4) helper moved to ring to play other roles (Fig. 4d, 14 screens, 10%). The first pattern was following role-filling individual rule, the third represent helping at the local subsystem and the second is intermediate (i.e. helper played the double-role). Although role-filling and role-switching are seemingly contradictory as a rule, they actually did because role-filling ranked higher than role-switching in the predetermined person-to-person defensive strategy. Moreover, the defensive team predeterminedly gradated role-switching criteria not to be predicted by the competitors (i.e. switch-recommended or avoided: see Supplementary Methods). The frequencies of transient help and role-switch were along with the favourable predetermined strategy (Fig. 4e: recommended role-switch was more than that in switch-avoided; in contrast, the relationship was reversed in transient help), suggesting that the helper selected desirable helping behaviour as a whole. We hypothesised that the helping behaviour at multiple subsystem scales depends on the context such as assumed emergency levels (Fig. 4e).

**Figure 5:**
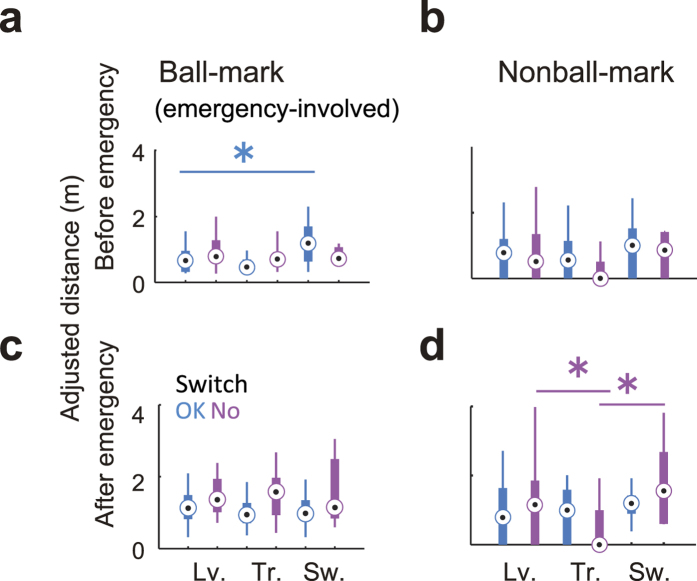
Helping behaviour characteristics. (**a–d**) Adjusted attacker-defender distance at two subsystem scales (**a**,**b**): Ball-nonmark distance and (**c**,**d**): Nonball-mark distance) before (**a**,**c**) and after (**b**,**d**) emergency involved with the emergency. Lv., Tr. and Sw. mean leave, transient and switch in emergency, respectively. Asterisk means significant difference between the patterns connected by the horizontal solid line. In recommended role-switch (Fig. 3e: 19 emergencies), the ball-mark distance involved with the emergency was larger than the leaving before the emergency (**a**) but not after the emergency (**c**). In switch-avoided situation after emergency (**d**), the transient help efficiently regulated the role-filling rule rather than the remaining patterns. The overall differences between before and after emergency (irrespective of the types of the helping behaviors) were shown in Fig. S2.

Helping patterns within a local subsystem emerged dependant on both the physical and strategic context. We first examined the adjusted roundabout distance (protuberance in Fig. S7a) of the helped during the screen as the competitor’s physical disturbance (see Supplementary Methods). Result demonstrated that in a role-switch in switch-avoided situation, the disturbance was larger than that in leaving (Fig. S7f, *F*_2,64_ = 3.17, *p* = 0.049, multi-comparison: *p* = 0.041), indicating strategically undesirable and physically emergency situation was occurred (Fig. 4e: only 6 emergencies). However, other coping behaviour was not explained by the physical disturbance. Regarding the degrees of threat, in more frequent recommended role-switch (Fig. 4e: 19 emergencies), the ball-mark spatial-gap involved with the emergency was larger than the leaving before the emergency (Fig. 5a: *F*_2,43_ = 4.45, *p* = 0.018, multi-comparison: *p* = 0.035) but not after the emergency (Fig. 5c, *p* > 0.05). The defensive subsystem intentionally may allow to stretch the spatial-gaps because role-switching can save moving distance for the helped (see Fig. 4c) and predictively avoid the competitor’s disturbance. In switch-avoided situation, the transient help efficiently regulated the role-filling rule rather than the remaining patterns (Fig. 5d: *F*_2,64_ = 4.97, *p* = 0.001, multi-comparison: both *p* < 0.045). This strategically desirable and high frequent role-overlapping helping behaviours (Fig. 4e: 24 emergencies) had the combined advantage of switching (avoid emergency) and leaving (follow the role-filling rule). Other subsystem behaviours, such as transient help in switch-recommended and leaving, did not show significant differences in physical disturbance and degrees of threat, suggesting that these subsystem behaviours emerged at lower emergency levels (Fig. 4e). All of subsystem behaviour requires close and efficient cooperation following primary and the lower-level rules in addition to predetermined rules, which seem to be a cascade reaction driven by the helper’s appropriate movement timing. In Suppelementary Text, we showed that the simple and earlier switching helping behaviour benefited on the system, but overlapped help timing depended on the situation.

### Helping behaviour at a global scale

**Figure 6:**
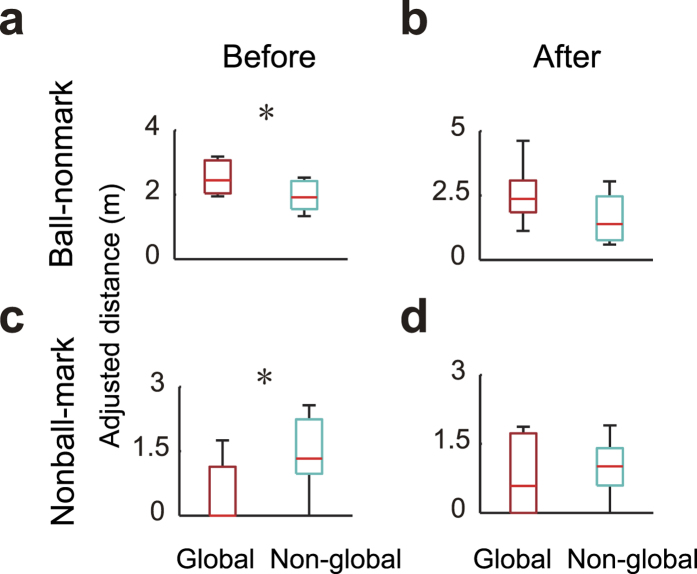
Global helping behaviour. Adjusted spatial-gap at 2 subsystem scales (**a**,**c**): Ball-nonmark, (**b**,**d**): Nonball-mark distance) of 5-on-5 system in global and non-global help before and after the emergency. Asterisk shows significant difference between global and non-global help. The ball-nonmark spatial-gap before the global help was larger than that before the non-global help (**a**), but not after the emergency (**b**), indicating that the coping with the first priority was required in global help and avoided the threat at a similar level to the non-global help. Conversely, the nonball-mark spatial-gap before the globally-helped emergency was smaller than that before the non-global help (**c**), but not after the emergency (**d**).

The helpers sometimes globally switched the action on the overall system for the most critical emergency of primary threat, not staying to help in the local emergency (Fig. 4d,e). Helper sometimes helped the primary emergency (i.e. shot) in 10 out of 14 ring helps (termed as global help: see Supplementary Methods). The ball-nonmark spatial-gap before the global help was larger than that before the non-global help (Fig. 6a, *t*_18_ = 2.4, *p* = 0.025), but not after the emergency (Fig. 6b, *p* > 0.05), indicating that the coping with the first priority was required in global help and avoided the threat at a similar level to the non-global help. Conversely, the nonball-mark spatial-gap before the globally-helped emergency was smaller than that before the non-global help (Fig. 6c, *W* = 78, *p* = 0.039), but not after the emergency (Fig. 6d, *p* > 0.05). Combined with the explicit signal of less disturbance of the screen (Fig. S14a, *p* = 0.026), the global help situation was not hazardous other than the ball and favourable condition of global helping. Additionally, the helper initiation timing at global help did not differ from other helping behaviour (Fig. S14b, *p* > 0.05), suggesting that the helper dealt with the two matters simultaneously rather than sequentially, from comprehensive perspectives. Although a screen-play represented an emergency to attract helper’s attention, too much attention to the emergency would make the uninvolved subsystem dangerous at a higher emergency level against another intended attack. Switching from the help in a localised space to the global help adapting the situation would be beneficial for the resilient system.

## Discussion

Although the defensive teams employed a simple rule to move to the ball and/or the marked attacker across the entire period, in emergency situations, the helper switched and overlapped the hierarchical subsystem (Figs 4, 5, 6). This role of the helper was merely one of the temporal roles in the overall system, however, it helped to create a resilient systems. First, at a present high emergency level, the helpers simply switched the local roles with the helped (Fig. 4c). Second, at an intermediate emergency level, the helpers effectively acted in overlapping subsystems (Figs 4b and 5d), and third, for the most critical emergency of primary threat, the helpers globally switched the action on the overall system (Figs 4d and 6). These behaviours benefited the entire group. Our results also suggest that the overall system explicitly understood^14^ and implicitly coordinated^16^ with the helper’s action at switched and overlapped subsystem scales, based on both top-down predetermined rules and real-time bottom-up shared intentionality^42^. This flexible and close coordination with minimised personal cost would be expected of flexibly role-switchable and organised efficient systems, rather than systems based on predetermined role-sharing^14^ such as non-human animals^20^,^22^,^43^ and human homogeneous joint actions^28^. We can therefore understand general principles that allow resilient systems to confront emergencies by switching and overlapping hierarchical subsystems.

Such resilient joint action would develop via communications with colleagues and competitors and adaptation to the circumstances. When helpers were helping their colleagues to cope with an emergency, they autonomously made decision based on the information about competitors in the heterogeneous field (Fig. 1c), and simultaneously obeyed the team rules that were dependent on the information held by colleagues (i.e. explicit instructions) and on the strategic context (Fig. 4e). Our results may be regarded as task- or system-specific; however, it is natural to assume that these cooperative helping behaviours^18^ would be learned and developed^44^ via explicit and implicit communications^42^ with colleagues (by creating pre-determined and implicit rules at hierarchical subsystem scales with teammates, coaches and tradition of the team and the game), and through competition against tactical competitors and adapting the heterogeneous circumstances. It has been claimed that only humans can have shared intentionality which has a recursive nature^42^ but only finite computations should be performed^45^ at multiple layers because of limitations on human cognitive resource. On this account, resilient joint actions will mutually develop in response to competitors and circumstances rather than emerge as complete solutions within the involved system. Creation, execution and evolution processes of helping behaviour in resilient systems should be further investigated.

Our results provide insights into social communication and the design of multi-agent autonomous systems. The term ‘resilience’ has been used in socio-ecological systems and in engineering^33^ but could be extended to social communication in human groups and adaptation within a living organism consisting of multiple subsystems. The resilience of social communication or individual adaptation can be estimated from the switching and overlapping in helping behaviour at appropriate subsystem scales, implying the local robustness of subsystem cooperation, or the reorganisation of global system against intended attack or unpredicted crises, which the previous framework could not explain. For example, modelling of cooperation^19^ such as polarised mobile agents^2^,^3^ cannot explain the switching hierarchical helping structure of resilient systems. Resilience in socio-ecological systems^46^ has been theorised as attractor dynamics^33^; however, asymmetric competition cannot be explained using this approach^47^. We expect that it will be possible to apply multi-layered helping behaviour to resilient cooperation in social organisms^2^,^3^,^26^ and human groups^1^,^12^,^41^ using mathematical simulations^10^,^11^,^23^. In practical terms, we can develop more resilient artificial autonomous systems such as multi-agent robots^6^,^7^,^8^,^9^ implementing these role-switchable and overlapping actions to manage risks such as recovery from system impairment.

## Methods

### Participants

Ten males from a top-level university basketball team in Japan (age = 19.5 ± 0.5 years, experience = 10.7 ± 2.4 years [mean ± SD]) participated in this study. The players provided their written informed consent to participate in this study. The experimental procedures were conducted in accordance with the Declaration of Helsinki and approved by the Local Ethics Committee in the Research Center of Health Physical Fitness and Sports, Nagoya University.

### Protocol

The players were divided into two teams (team A and B) and played 5-on-5 half-court (14 m × 15 m) basketball game alternately as an offensive team to shoot the ball within 20 s and then as a defensive team. Each trial began at the initial position fixed by the coach with a pass from the defender to the dribbler, and it finished with a successful shot, rebound by a defender or turnover (i.e., out of bounds of the court or a defensive team holding the ball). In the event of a defensive foul or out of bounds, the same offensive team started attacking again with the ball (i.e., reset trial which was excluded from the analysis). The game was played with two referees according to the rules of basketball except for the specific rules stated above. The total number of 5-on-5 games (trials) was 73, which comprised 31 successful shots, 35 defensive rebounds or turnovers, and 7 reset trials. We analysed 54 trials, including 61 attacks (26 successful shots, 26 failed shots and 9 ball-lost) in which screening play was detected in the way described below (screen feinting was not also detected as screen in this study).

### Measurement

Three-dimensional coordinates of the landmark points were acquired using a 3D optical motion capture system with 6 cameras operating at 100 Hz (OptiTrack Prime W17, NaturalPoint, USA). Reflective markers were placed on each player’s body (top and left of the head and right and left side of their shoulders). We used the midpoint of shoulders as the representative point of the players. We also used the basketball pasted reflective sheet in a striped pattern to improve ball handling and recorded its position. All raw coordinate data points were smoothed using a fourth-order Butterworth low-pass digital filter (6 Hz). All numerical calculation was performed using the MATLAB 2011a Statistical Toolbox (The MathWorks, Inc., MA, USA).

### Scene selection

We constructed automatic play-detection system using the positional data (for detail, see Supplementary Methods). First, we categorized the state of the ball into holding, passing and shooting. We analysed the duration before the attacker’s shot or ball lost. To analyse emergent subsystem behaviour, we then computationally detected the defenders who marked each attacker, the state of the ball and the screening behaviour of players (for detail, see Supplementary Methods).

### Attacker-defender distance as degrees of threats

We considered adjusted attacker-defender distances reflecting the spatial and predictive specificity of the environment. A desired defensive position was defined as a position desired defensive distance (0.5 m) from the attacker’s position in a straight line connecting the attacker’s position and the ring. The attacker-defender distances were calculated in four ways (Fig. S1a, Fig. S1 and Video S1): (1) Euclidean distance from the desired defensive position to the current defender’s position; (2) spatially-corrected distance, i.e., the Euclidean distance multiplied by the successful shot probability calculated by the linear regression of the distance between the shot position and the ring (Fig. 1d); (3) prediction-corrected distance, i.e., the Euclidean distance corrected by the time required to catch the ball passed by another attacker and the distance that the attacker can move during the ball was passed based on the attacker’s current velocity, and (4) the spatially- and prediction-corrected distance. Time series of adjusted distances were not smooth at the start of the pass because attacker with the ball abruptly switched, thus, we eliminated the instant from calculation (Fig. S1a).

To take the effect of screening on the predicted distance into consideration, we defined the roundabout distance of the user-defender during the screen as the protuberance (Fig. S7a; maximum 1 m), which was obtained by the perpendicular line from the current screener’s position to a line connecting the user-defender’s desired and current position (we termed the Euclid roundabout distance). We assumed the user-defender moves parallel to the protuberance, and subsequently toward the desired position (trajectory of rectangular triangle in Fig. S7a). During screening, we calculated the predicted distance based on the trajectory.

To examine characteristics of attacker-defender distance at multiple subsystem scales, we exclusively categorised 25 attacker-defender distances (5 attackers × 5 defenders) into 4 distances (Fig. S1b and Video S2), which were analysed as the maximum values of the attacker-defender distances: (1) maximum ball-mark distance, i.e., the maximum distance between the attacker with the ball and the defender marking the attacker; (2) max-min ball-nonmark distance, i.e., the maximum distance between the attacker with the ball and the nearest defender to the attacker other than the defender marking the attacker; (3) maximum nonball-mark distance, i.e., the maximum distance between the attackers and the defender marking each attacker other than the ball-mark distance, and (4) max-min nonball-nonmark distance, i.e., the maximum distance between the attackers and the nearest defender to each attacker after removing the above 3 distances. The 4 × 4 maximum distances (four correction methods and four subsystem scales) in specific interval were used in analyses below.

### Defender’s coping behaviour with screen

We computationally detected defender’s coping behaviour with screen (see Supplementary Methods). We then categorised the screen coping behaviour as a dyad into 4 patterns: leaving, transient help, role-switch and ring help (in detail, see Supplementary Methods).

To consider the context in team strategy shared in advance, we further categorised the switching and transient help into those in switch-recommended and switch-avoided strategy. In switch-recommended strategy, switching help was recommended in the team defence in the specific pair of defender (such as pair of guard) or in specific situation (specific offense formation), according to the head coach of the team. If the situation other than the above, we defined it as the switch-avoided situation, taking priority of person-to-person defence rule.

### Analysis of cooperating behaviour at multiple subsystem scales

Similar in the attacker-defender distance, we calculated four maximum roundabout distances (i.e. Euclid, spatially-corrected, prediction-corrected and both-corrected) corrected by shot probability and/or distance between attacker with ball and user-defender (Fig. S7b) as an index of the effectiveness of the screening.

For comparison with variables such as maximum roundabout distance and various initiation times and for examining correlation with categorisation such as coping subsystem behaviour with screen, the adjusted attacker-defender distances involved/uninvolved with and before/after the screen were calculated at focused subsystem scales. The distance involved with the screen was calculated using 4 distances (2 attackers and 2 defenders involved with the screen) with the same algorithm and the uninvolved was calculated using 9 distances (3 attackers and 2 defenders uninvolved with the screen). The distance before and after the screen were estimated using the interval from 5 s before the start of the screen until the start, and before the end of the screen until 5 s after the end, respectively, considering the hysteretic effect and cause of the screen.

### Statistical analyses

The probability of the successful shot was linearly regressed with the distance from the ring. *F*-value and *R*^2^-value were calculated as test for the significance and the contribution in the regression, respectively. Comparison between various distances and the outcome of defence (success was attacker’s failure of shot and loss the ball) were performed using logistic regression analysis. The Nagelkerke *R*^2^ as a coefficient of determination describes which fraction of the variability is explained. Hosmer-Lemeshow goodness-of-fit chi square to study the calibration of the models. AIC (Akaike information criterion) was used to compare the goodness-of-fit of the model. We did not used multi-regression analysis because the explanation variables such as the various distances were in different contextually- or spatially-hierarchies and we should separately examine these variables.

To compare the variables in various defender’s coping behaviour with the screen, one-way ANOVAs (*F*-value was calculated for significance) were used with the coping behaviour (different strategy were separately analysed because of contextually-different situation), if the hypothesis of homogeneity of variances between systems was accepted with Bartlett’s test. If rejected, the Kruskal-Wallis nonparametric tests were performed to compare these variables. An unpaired *t*-test or Wilcoxon rank sum test with Bonferroni correction (*t*-value and *W*-value was calculated, respectively) was used to compare the variables within the factor where a significant interaction in ANOVA or a significant effect in Kruskal-Wallis test was found, respectively.

For comparison of variables between the global help and the non-global help behaviour, we used unpaired *t*-test if the normality assumption was accepted by Lilliefors Test. If rejected, Wilcoxon rank sum test was used. For all the statistical calculations, using p-value, *p* < 0.05 was considered significant. Statistical analyses were performed using the MATLAB 2011a Statistical Toolbox (The MathWorks, Inc., MA, USA) and PASW Statistics (version 18.0.0, SPSS Inc., Chicago, IL, USA).

## Additional Information

**How to cite this article**: Fujii, K. *et al*. Resilient help to switch and overlap hierarchical subsystems in a small human group. *Sci. Rep.* **6**, 23911; doi: 10.1038/srep23911 (2016).

## Supplementary Material

Supplementary Movie 1

Supplementary Movie 2

Supplementary Movie 3

Supplementary Movie 4

Supplementary Movie 5

Supplementary Movie 6

Supplementary Movie 7

Supplementary Information
