# Supplementary material for: Resilient help to switch and overlap hierarchical subsystems in a small human group
Source: Sci Rep. 2016 Apr 5;6:23911. doi: 10.1038/srep23911 (PMC4820690; doi:10.1038/srep23911)
Supplement: Supplementary Information [file srep23911-s8.pdf]

Supplementary materials for:

Resilient help to switch and overlap hierarchical subsystems in a small human group

K. Fujii, K. Yokoyama, T. Koyama, A. Rikukawa, H. Yamada, Y. Yamamoto

This PDF file includes:

1. Supplementary Methods

2. Supplementary Text

3. Figure S1 to S15

4. Tables S1 to S2

5. Legends of supplementary videos S1 to S7

Methods

Scene selection. We constructed automatic play-detection system using the positional data (parameter values were shown in Table S1). First, we categorized the state of the ball into holding, passing and shooting. (1) If the ball and an attacker was near (horizontal distance was less than 1 m) and horizontal ball acceleration was less than –5 m/s^2 during ball catch (i.e. the ball was in the air), the state of the ball was defined as the holding. The involved attacker was defined as the ball attacker. (2) If the ball was not holding, ball vertical position was adequately above the ring (more than 3.2 m: the ring height was 3.05 m) and the ball was horizontally near the ring (less than 0.5 m: the ring diameter was 0.45 m), the ball was detected as a shot. (3) If the ball was neither holding nor shot, the ball was passing because the trial was ended when a defender hold the ball. We analysed the duration before the attacker's shot or ball lost. When the attacker gain the ball after the shot (i.e. offensive rebound), if the ball was brought out of the remote place from the ring (more than 5 m), we analysed the attacks aside from the attacks before the shot.

To analyse emergent subsystem behaviour, we computationally detected the defenders who marked each attacker, the state of the ball and the screening behaviour of players. The defender marking each attacker was defined as the defender nearest in terms of the Euclidean distance to the attacker at the start of the trial. We then defined a

‘switching behaviour’ (position was shown in Fig. S3b) where one defensive player switched marking an attacker with a teammate, as the behaviour where both distances between the two defenders and opposite marking attackers were shorter than the ordinary marked attacker-defender distance, and the distance between the two attackers was more than a switch threshold (2 m). The state of the ball was defined as follows: (1) If the ball was near to an attacker (within 1 m), the state of the ball was held by the attacker. The accurate times of ball catching and release were calculated based on the acceleration of the ball. (2) If the ball was in the air and moving toward the ring, the ball state was detected as shooting. (3) If the ball was not held nor shot, the ball state was passed.

Screening behaviour aims to disrupt the advance of a defensive player (termed user-defender or the helped) from moving freely toward the marked attacker using the screen (termed user) by legally blocking another attacker (termed screener). The screening was defined as a behaviour where the screener approached the non-marked, user-defender (who did not mark the screener) within 1 m and both attackers then approached within 1 m and within 2 s (position was shown in Fig. S3c-d). The screening interval was ended when the defenders completed marking each attacker after switching 0–2 times. For analyses below, we regarded the screen play involved with more than 3 attackers also as the last 2 attackers’ play involved with the screen. We focused on the screening behaviour of attackers and analysed the interval from the start

of the first screen to the start of a shot or turnover.

Selection of defender's coping behaviour with screen. The defender's initiation time was estimated by the peak accelerated moving acceleration (Fig. S4a). First, we detected the defender's horizontal peak acceleration using MATLAB function 'findpeaks', in which minimum peak distance was set at 4 m/s^2 and minimum peak interval was set at 0.1 s. The former threshold was determined by minimum value of each player's horizontal peak acceleration more than 80 % frequency during whole playing time. The latter threshold was determined assuming that one movement initiation (i.e. movement of centre of mass) takes at least 0.1 s during ballgames (Fig. S4b). Second, because the peak horizontal acceleration itself means acceleration and deceleration and there was no relationship between horizontal velocity, we selected the peak accelerated moving acceleration defined as the peak acceleration which satisfied that dot product of unit vector of acceleration and velocity at the same time was over zero and the velocity was over 1 m/s during the time from the peak acceleration and the drop to the minimum peak acceleration threshold（Fig. S4b）.

To evaluate the behaviour of individual defenders during screening, we then used the dot product of the defender's maximum horizontal velocity during the time from the peak acceleration and the drop to the minimum peak acceleration threshold, and the unit vector from the current to the desired defender's position against the target attacker (i.e., user or screener) or ring to help other teammate than players involved

screening. For user-defender, if the user-defender moves toward user or parallel to user (i.e. dot product of the user-defender's horizontal velocity and position from future user-defender to user or velocity vector of user was more than that of position vector from user-defender and screener, respectively), the user-defender's behaviour was detected as moving toward user. If not, the direction of user-defender was detected as the executing action involved screener except for stepping away from screener (in this case, we defined it as other action). We simplified the various emergent actions of user-defender involved screener, such as switching behaviour from leftward or rightward of screener. For screener-defender (Fig. S5), we first separated situations whether the user positioned at the ring or not with threshold (3 m). The former case means screen was occurred near the ring (i.e. more emergent screen) and there was little possibility of other actions than movement to user, screener, and the ring (e.g. cross screen called in basketball). If latter, we additionally separated situations whether the user approached to the ring or not with threshold (within 90 degrees of velocity direction). The former means the direction of the ring and user velocity was near (e.g. back screen called in basketball), thus, screener-defender's movement to the ring was also defined as the movement to the user. In all cases, the direction of screener-defender was detected as the behaviour involved with user if screener-defender moves toward user or parallel to user (i.e. dot product of the screener-defender's horizontal velocity and position from future screener-defender to user or velocity vector of user was more

than that of position vector from screener-defender and screener, respectively). Similarly, the second priority of detection was the behaviour involved with screener if screener-defender moves toward screener or parallel to screener. In the case near the ring, if screener-defender did not move to user nor screener, we defined as the movement involved with the ring (e.g., help teammates other than user-defender). If the user did not approach the ring and was distant from the ring and the screener-defender moved toward the ring, the behaviour was detected to move toward the ring. In the remaining case, the behaviour was determined as the other behaviour.

Candidate optimal timing of initiation of helping behaviour. We calculated the screener-defender's candidate optimal initiation timing toward user to help the user-defender (Fig. S8). We proposed two candidate optimal timing to minimize two attacker-defender distance as assumed related cue. We assumed that if screener-defender initiates toward user in the most guardable situation, the screener-defender can help the user-defender. First, we propose the simplest cue based on the distance between user and screener-defender. Second, we used the remaining distances i.e. the maximum distance between minimum distance between user-and-user-defender distance and screener-and-user-defender distance, and screener-and-screener-defender distance (Fig. S8). To examine correlation relationship between variables, we used Pearson's correlation coefficient was used if the normality assumption was accepted by Lilliefors test. If rejected, we used the Spearman's rank

correlation.

2. Supplementary Text

1.1 Degree of threat at multiple subsystem scales in heterogeneous field

The directly simplest distance to foil a shot was considered to be the Euclid distance between the attacker with a ball and the defender marking the player (Euclid ball-mark distance: Fig. 2-3). However, the maximum Euclid ball-mark distances in failed defence were not significantly different from those in successful defence (Fig. 2a, all $p > 0.05$). Instead, we examined distances with correction of static-spatial specificity and dynamic-predictive specificity at multiple subsystem scales. The maximum distances with the contextual-heterogeneity correction at three different subsystem scales (Fig. 2d and Table S2: ball-mark, ball-nonmark and nonball-mark) and ball-mark distance with static-spatial correction in successful defence were larger than those in failed defence (Fig. 2b and Table S2: all $p < 0.29$ and odds ratio > 2.98). For example, if distance between the attacker with a ball and the defender marking the player (ball-mark distance) with the correction of shot success probability and pass and moving prediction (i.e. contextually-heterogeneous correction), the large maximum ball-mark distance explained successful defence ($p = 0.012$), and the odds ratio was 6.4 (95 percent

confidence interval was 1.5 to 26.9), meaning that the defence was 6.4 times more likely to succeed if the maximal adjusted ball-mark distance decreased by 1 m. Remaining statistical values are shown Table S2. Furthermore, we analysed in immediate-before shot interval (during 1 or 2 players holding the ball) and demonstrated that results of the distance involved with attacker with the ball was similar (Fig. S9a-d upper and Table S2); however, those uninvolved with the ball were not different between successful and failed shot (Fig. S9a-d lower, all $p > 0.05$). The spatial-gaps immediately-before the shot involved with the ball are assumed to be direct cause of the successful shot probability. On the other hand, those in entire period of analysis uninvolved with the ball, which indicates the degree of following the defensive team rule, would be remote cause of the probability in different time scale.

Our results indicated that in the field with contextual heterogeneity, the critical competitive inter-agent distance should be adjusted by the static spatial specificity (targets: goal and lines) and dynamical predictive specificity (signals: ball and players position and velocity), evolving through massive amount of communication such as competition, cooperation or being coached. For example, in less experienced groups the critical distance may be primitive similarly to Euclid distance, and in another sophisticated group the critical distance might be more complicated. Although in this analysis agents were homogeneous within a group, our methods can reflect the individual variation such as more precise spatial specificity in successful action

probability or speed of action or another-level contextual information such as remaining time.

1.2 Emergent helping behaviour in a local subsystem

With respect to the adjusted attacker-defender distance before and after the screen-play, most of adjusted distances for the uninvolved players and the uninvolved players were not different between success and failure before and after the screen play (Fig. S2a,c-d). However, the adjusted ball-mark distance for the involved players after screen in successful goal was larger than those in failed (Fig. S2b and Table S2: $p = 0.008$ and odds ratio was 5.34).

Two adjusted distances in leaving screen in switch-avoided situation for the uninvolved players before the screen were larger than that in other coping behaviour (Fig. S10b, both $p < 0.05$), suggesting that leaving the emergency in relatively more choice might cause another crisis.

1.3 Helping behaviour timing in a local subsystem

The simple and earlier switching helping behaviour benefited on the system, but overlapped help timing depended on the situation. The helper initiation timing relative to the start time of the emergency (i.e. when the screener and user-defender approached) as a tactical event had no relationships with the degrees of threat (Fig. S11,

all p > 0.05). However, initiation time relative to the timing when the helper and the direct target (i.e. screen-user) was the nearest showed some thought-provoking results (other timing was explained below). Although the helper's relative initiation timing did not affect the following involved subsystem spatial-gaps (Fig. S12b, all $p > 0.05$), in recommended role-switch, the earlier helper acted, the smaller the distance of the uninvolved spatial-gaps became (Fig. 12d blue left bottom, $r_{11} = 0.67$, $p = 0.013$). Thus, early role-switch benefited on the system possibly because applying the predetermined role-switching strategy can prepare the uninvolved subsystem for subsequent movement, but a later switch may confuse the uninvolved and might drive the uninvolved helpers to the involved. Regarding transient help in a switch-avoided, the earlier the help initiated, the larger the uninvolved spatial-gap became (Fig. 12d red right bottom, $r_{22} = –0.50$, $p = 0.013$). The reverse result occurred because the larger involved spatial-gap before the emergency caused earlier help (Fig. 12a red right bottom, $r_{22} = –0.46$, $p = 0.024$), which indicates that a larger emergency was occurred before the screen. In other words, in contrast to the recommended role-switch, the solution of a transient helper's engagement timing for an overlapped goal with role-switch and role-filling would depended on other factors such as local competitive interaction[47] in which both mutually quick decision making and execution are required. Furthermore, in transient help in switch-recommended, more complicated result was observed. In this situation, the relatively-earlier or later helper initiated, the smaller the uninvolved distance was

(Fig. S12d right blue bottom, $r_8 = –0.71$, $p = 0.020$), indicating more complicated result seemingly mixed with the results of switch-recommended role-switch and switch-avoided transient help. These results suggest that the transient help requires more vigilant coordination in the subsystem than role-switch depended on the complex context to cope with the emergency. In a present high emergency level, the earlier helping behaviour in recommended role-switch benefited on the system, but in an intermediate level, the more complex helping at overlapped subsystem did not show that the earlier was the best.

Other candidate relative timing, which was minimum timing of maximum distance between screener-screener-defender and minimum of user-user-defender and screener-user-defender, was examined (Fig. S13). Results showed that there were no relationships between them other than the nonball-mark distance in transient help after the screen uninvolved with screen (Fig. S13d right blue bottom, $r_8 = –0.73$, $p = 0.016$). The significant correlation was similar to Fig. S12d. We also examined another timings, this result was robustly observed (minimum timings of various distance were similar). The results that there were similar or no relationship with spatial-gaps, indicate that the relationship between the helper and direct target may be critical in the helper's decision to help.

1.4 Emergent helping behaviour at a global scale

Ball-mark spatial-gap in before and after globally-helped crisis was not different from that in non-global help (Fig. S14c,d: $p > 0.05$). The nonball-nonmark spatial gap showed similar results of the nonball-mark spatial gap (Fig. S14e,f).

3. Supplementary figures

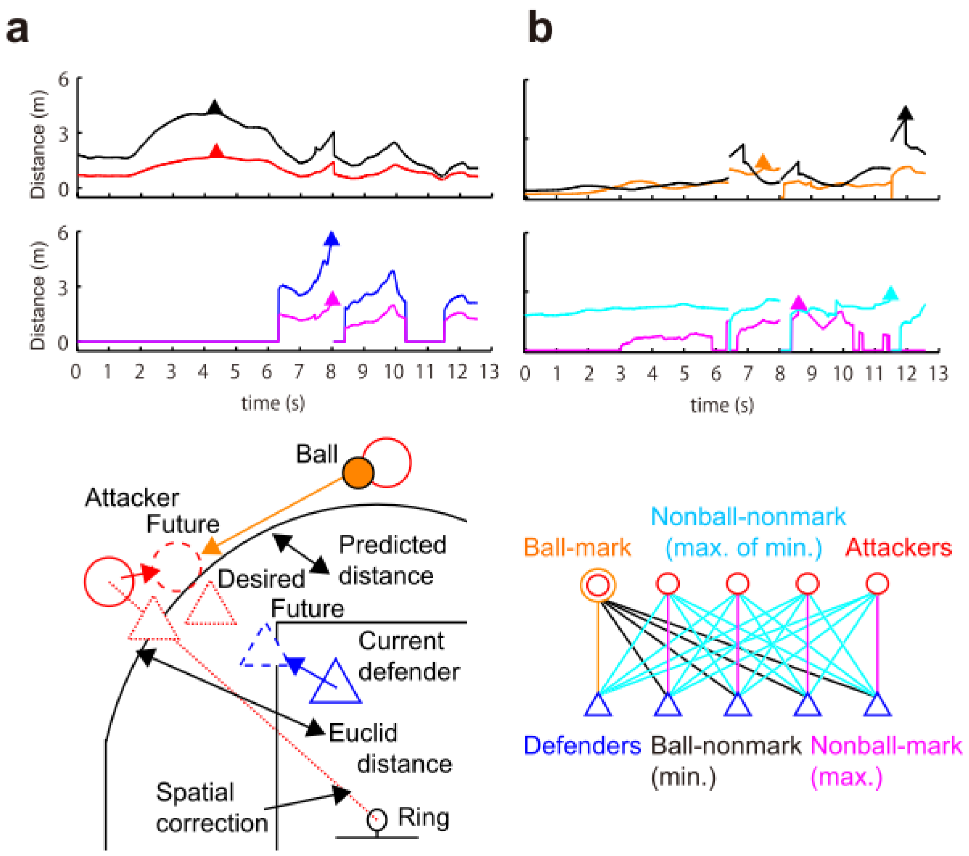

Figure S1. Examples of attacker-defender distances. (**a**) Examples of time series of 4 kinds of the distance adjusted by the heterogeneities. (**b**) Examples of the time series of the distance at 4 subsystem scales. For details on the two diagrams (**a-b**), see Fig. 2.

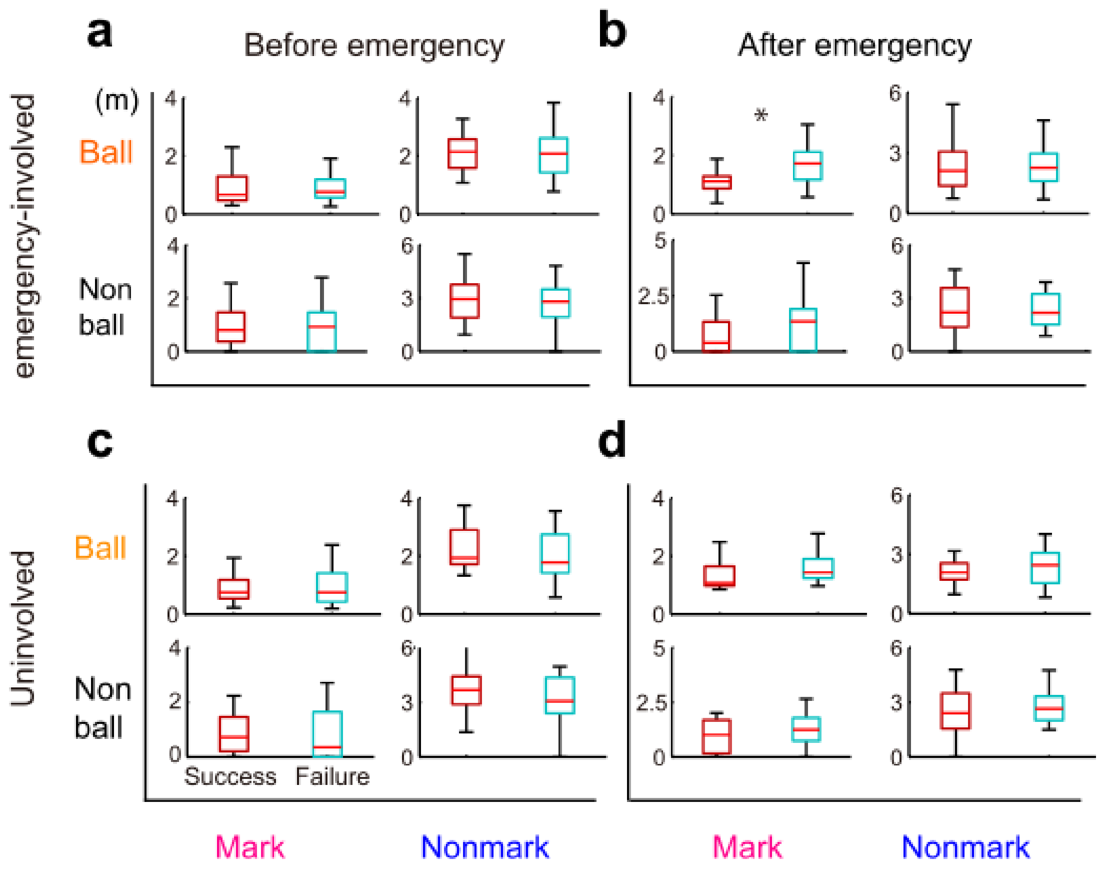

Figure S2. Maximal adjusted distance involved with screen. The maximum adjusted distance involved (**a-b**) and uninvolved (**c-d**) with screen before and after the screen at 4 subsystem scales. Configuration and sign are the same as Figure 2.

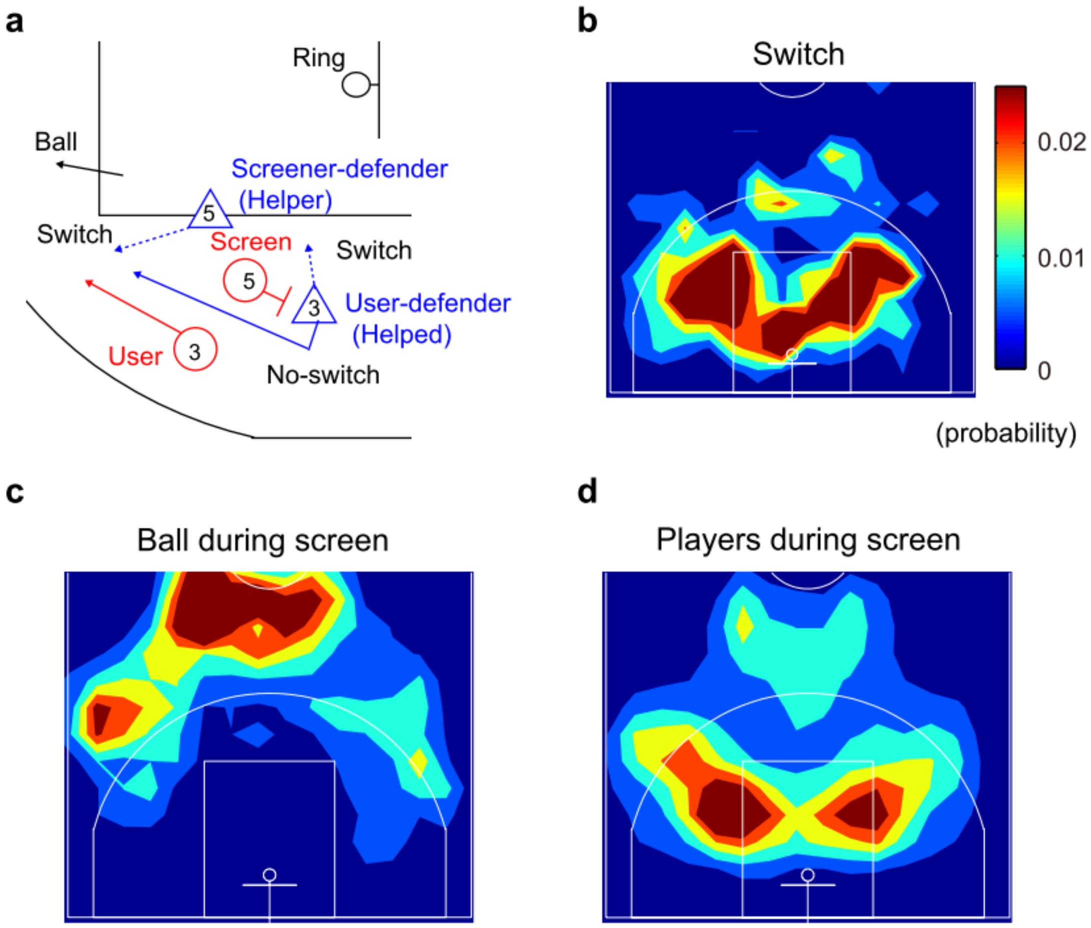

Figure S3. Switch and screen. (**a**) Diagram of switch and screen. Initially, user-defender (blue 3: helped) and screener-defender (blue 5: helper) marked user (red 3) and screener (red 5) as defined, respectively. If switching, user-defender and screener-defender marked screener and user, respectively. Switch can shorten the moving distance of user-defender, but cooperative movement between user-defender and screener-defender. (**b**) Heat map of 4 players position (2 attackers and 2 defenders) when switching. (**c-d**) Heat map of position of ball (**c**) and all players (**d**) during screen.

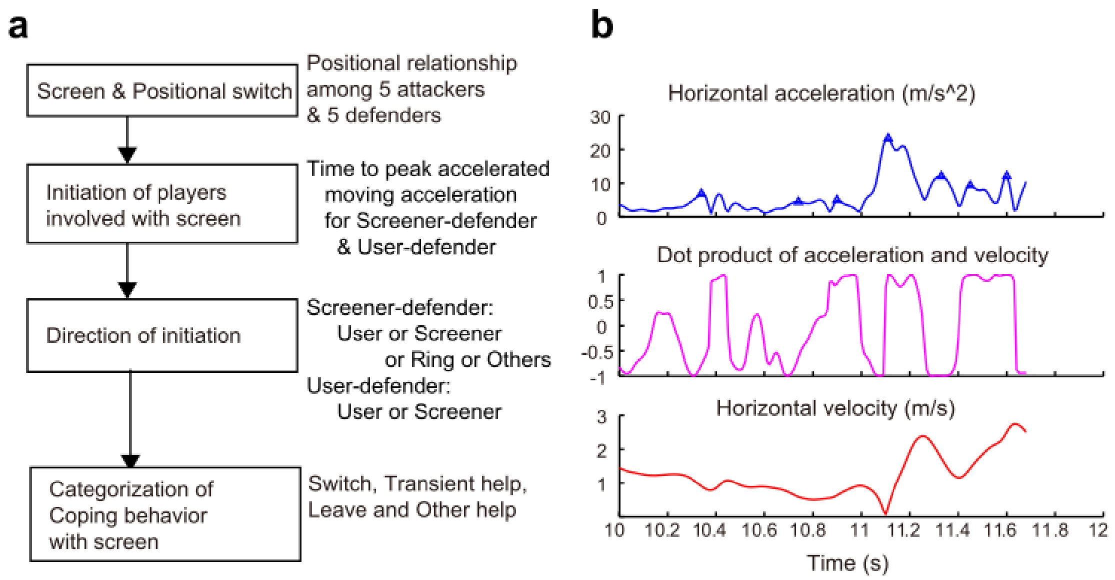

Figure S4. Direction of defender's behaviour. (**a**) Procedure of categorisation. (**b**) Examples of defender's horizontal acceleration (top), dot product of horizontal velocity and acceleration (middle) and horizontal velocity (bottom). Triangles show peak accelerated moving acceleration as initiation time (see Supplementary Methods).

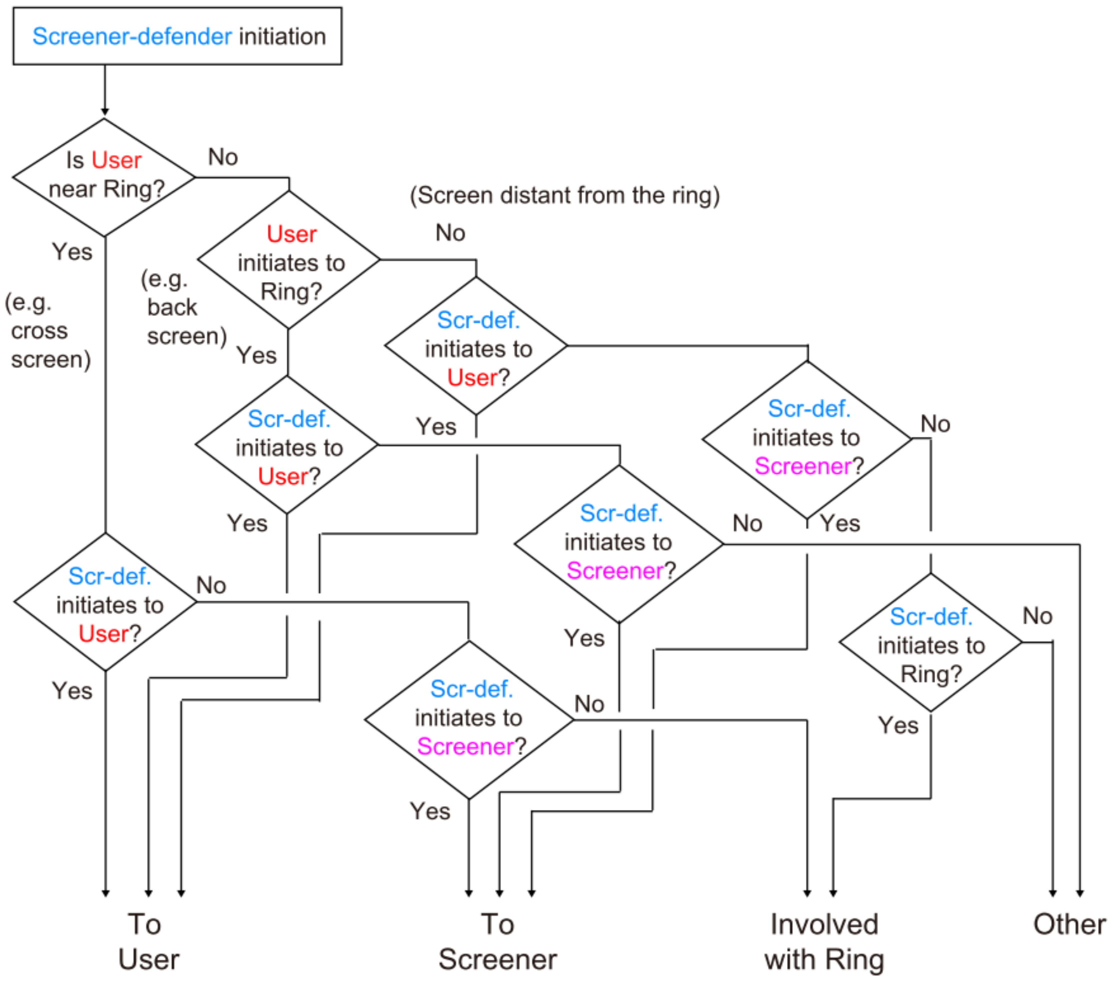

Figure S5. Block diagram of categorisation algorithm of screener-defender's behaviour.

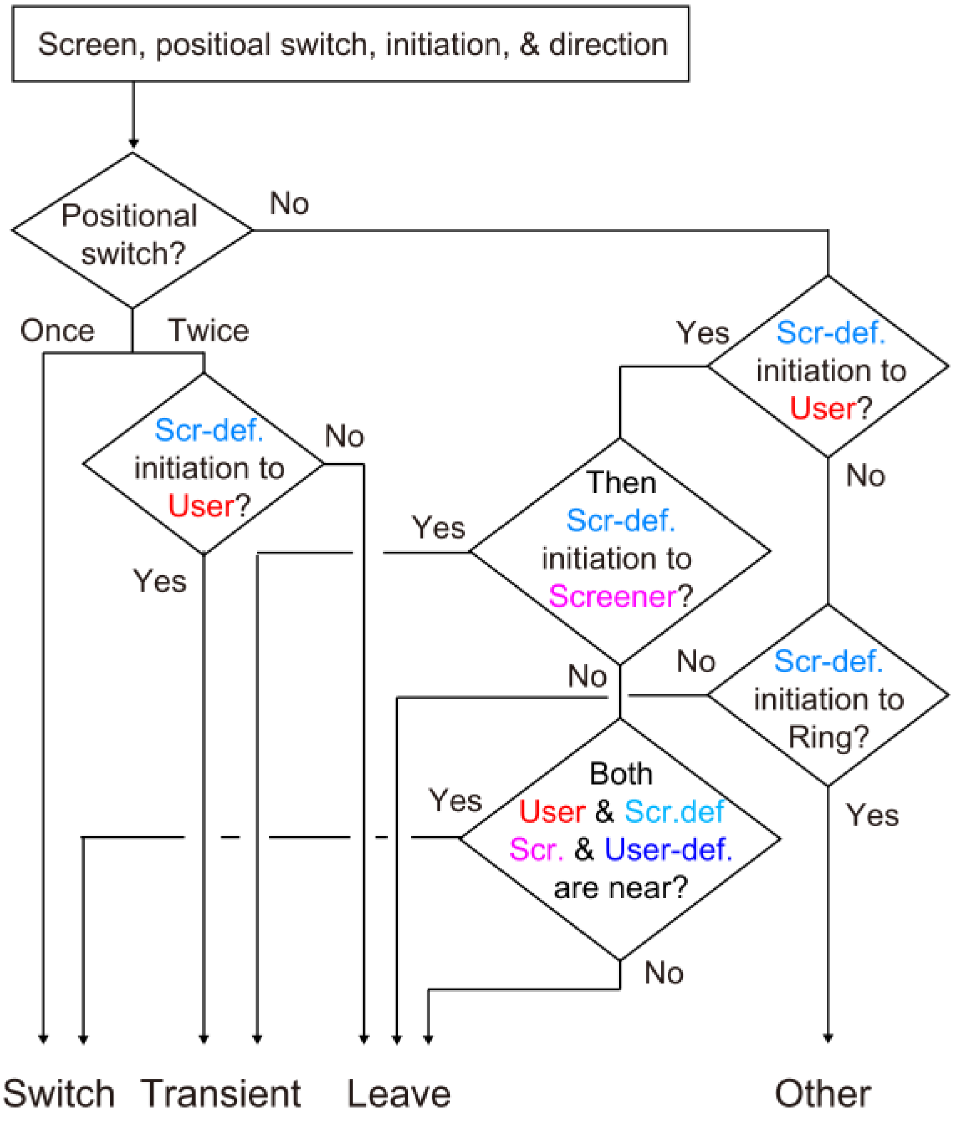

Figure S6. Block diagram of categorisation algorithm of defenders helping behaviour during screen.

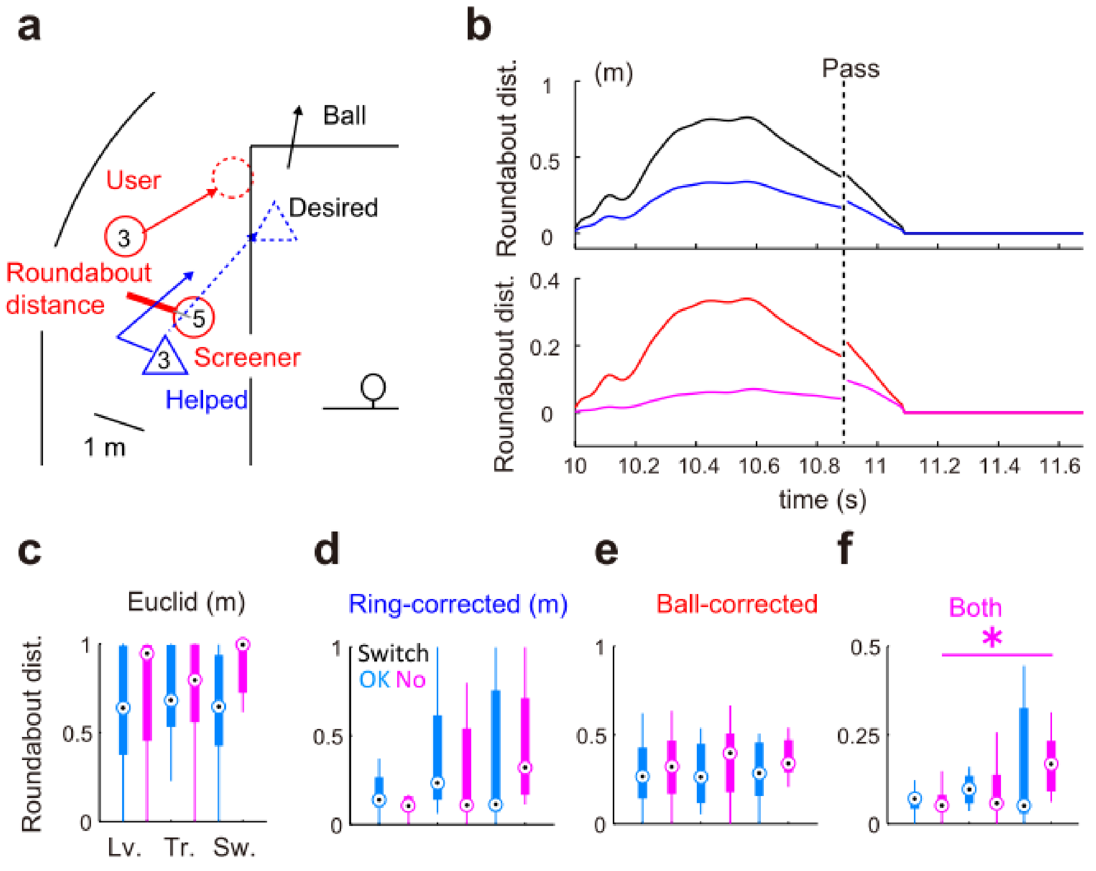

Figure S7. Roundabout distances. (**a**) Diagram of roundabout distance (red thick line) as physical indicator of emergency. Calculation methods are shown in Supplementary Methods. (**b**) Examples of the roundabout distance corrected by successful shot probability with distance from ring (Fig.1d: ring-correction) and/or the distance between the user-defender and the ball (ball-correction): Euclid (black), ring-corrected (blue), ball-corrected (red) and both-corrected (magenta) roundabout distance. (**c-f**) Four adjusted roundabout distance among three major cooperative patterns (switch, transient and leaving) and two pre-determined strategies (switch-recommended and switch-avoided).

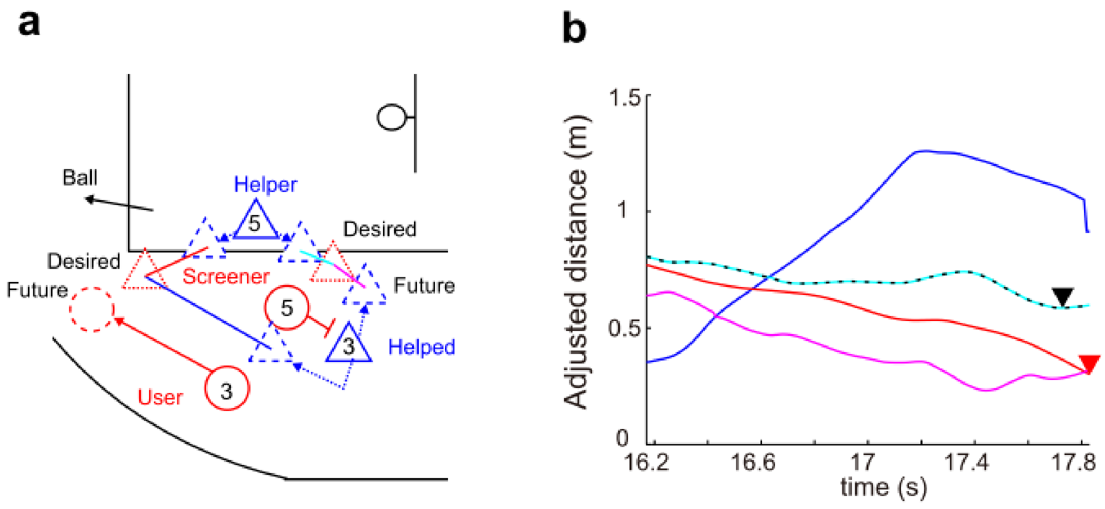

Figure S8. Candidate helper's optimal initiation timing. (**a**) Diagram of 4 kinds of attacker-defender distance. (**b**) From 4 kinds of distance (same colour as A), we examined 2 distances as candidate helper's optimal initiation timing: (1) user and screener-defender distance (red line) and (2) remaining distance (black dashed line, see Supplementary Methods). Triangles are minimum distances used to determine candidate helper's optimal initiation timing.

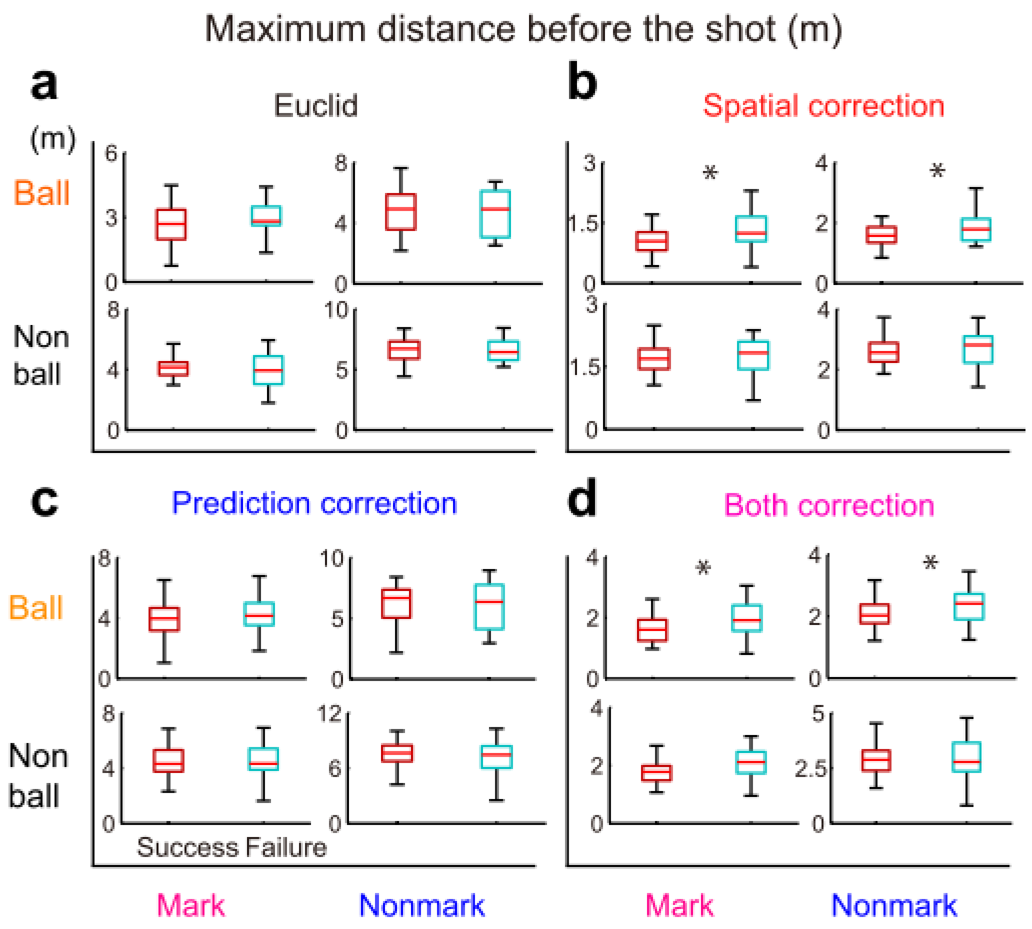

Figure S9. Maximal adjusted distance before the shot. The 4 × 4 distance (corrections and subsystem scales) in successful (red) and failed (cyan) shot immediately before the shot. Asterisk shows significant difference between the distance in successful and failed defences.

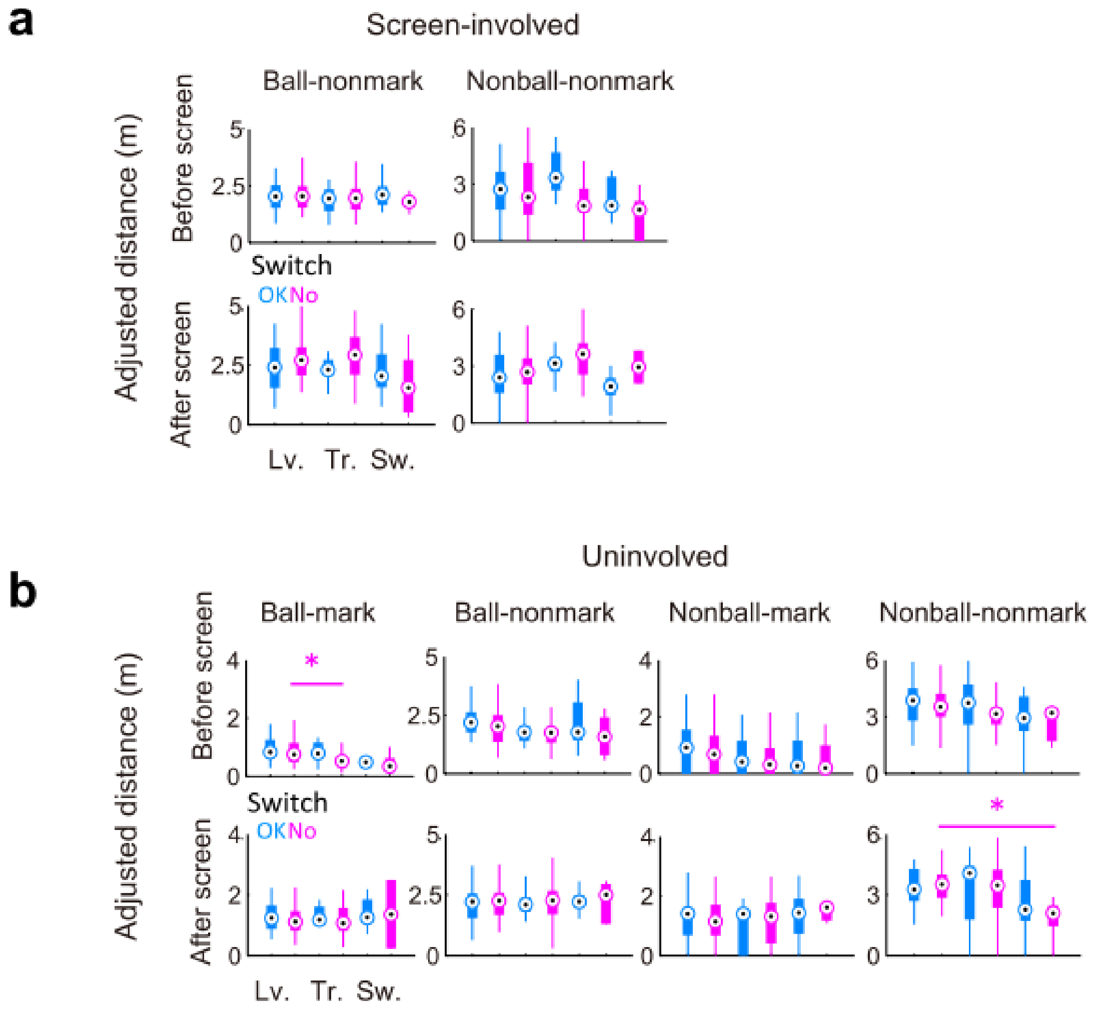

Figure S10. Maximal adjusted distance in various helping. The maximum adjusted distance involved (**a**: remaining scales in Fig. 5a-d) and uninvolved (**b**) with screen at 4 subsystem scales. Configuration and sign are the same as Fig. 5.

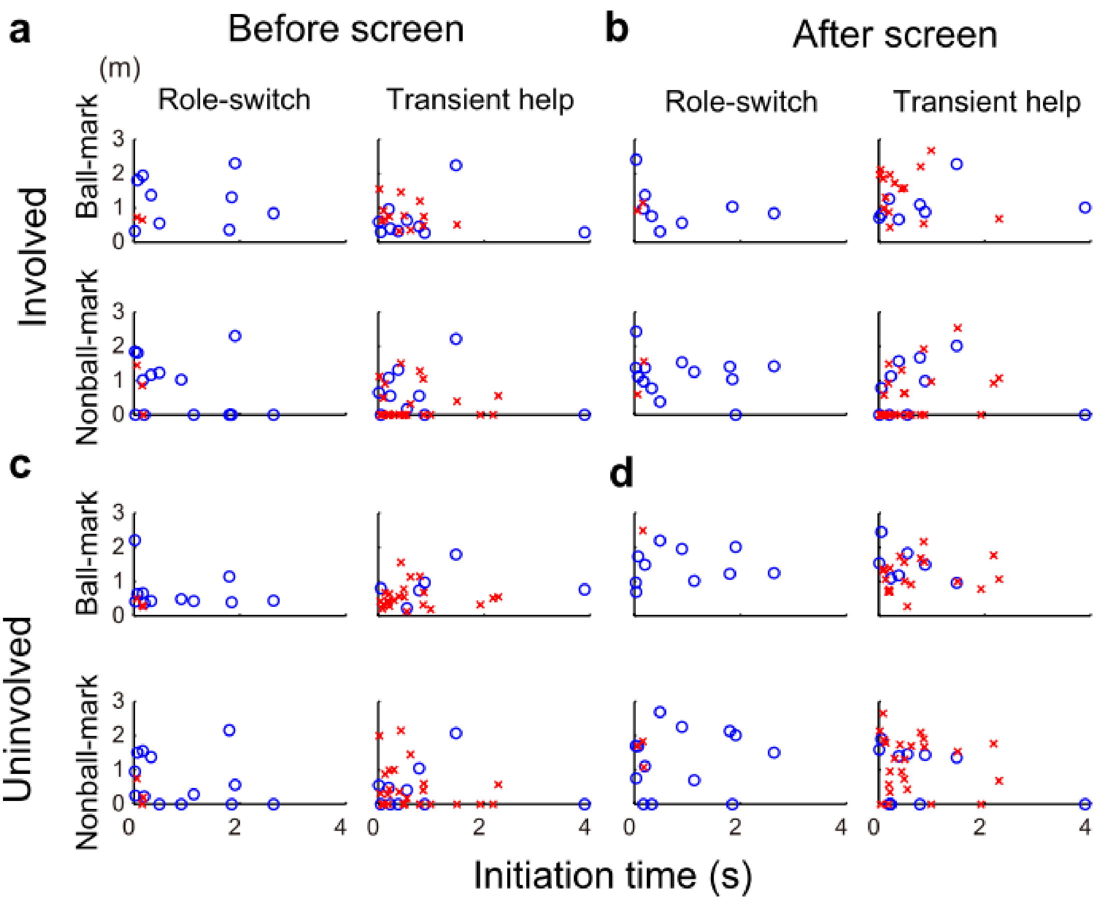

Figure S11. Helper's initiation and adjusted distance. All plots show relationships between helper's initiation timing relative to screen start and adjusted spatial-gap involved (**a,b**) and uninvolved (**c,d**) with and before and after the screen. Blue circle and red cross means switch-recommended and switch-avoided situation, respectively.

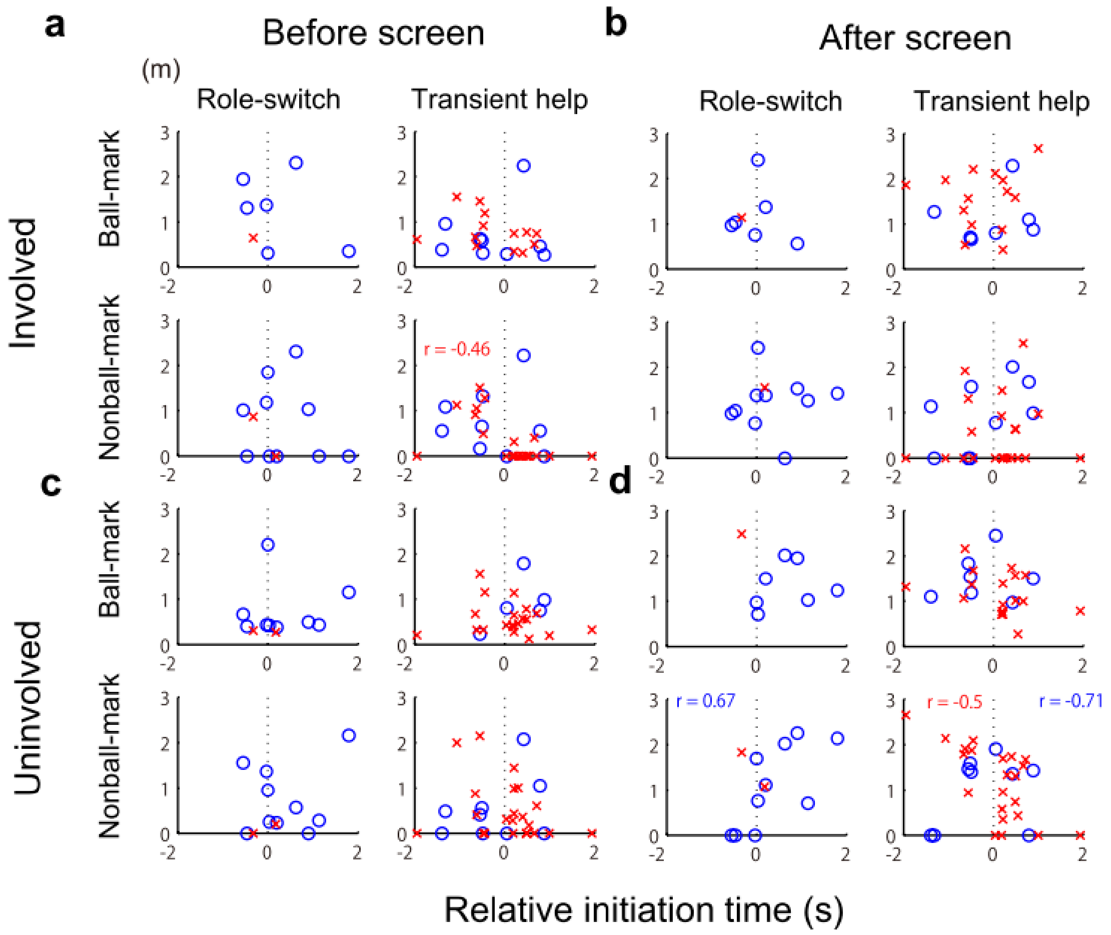

Figure S12. Helper's relative initiation timing and adjusted distance. Configurations are the same as Fig. S11. All plots show relationships between helper's relative initiation timing to minimum distance timing between user and screener-defender, and 2 adjusted spatial-gaps. Figure 6e-h is the part of (**c-d**). R values (colours correspond with the strategies) are shown if the correlation was significant (left and right are correlation coefficients with calculated and absolute relative initiation timing, respectively).

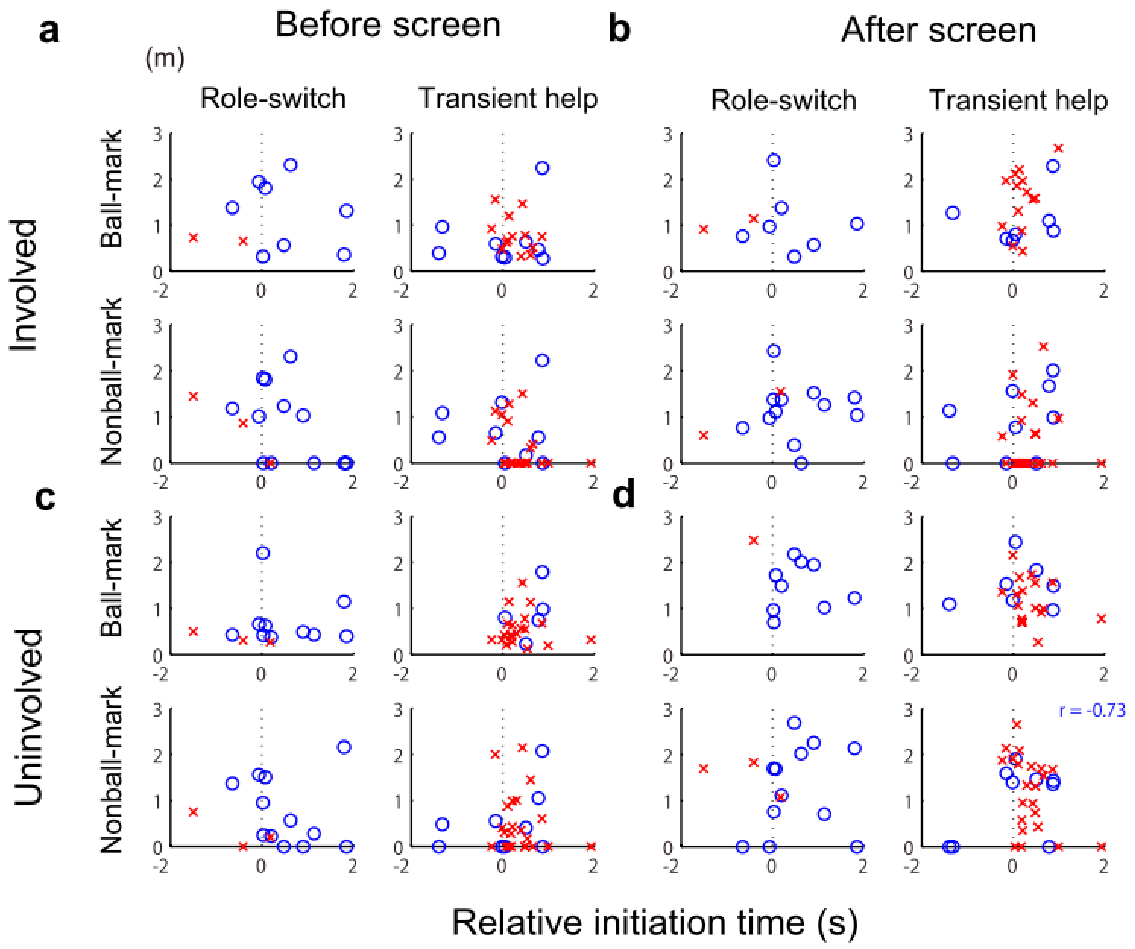

Figure S13. Helper's other relative initiation timing and adjusted distance. Configurations are the same as Fig. S12. As another candidate relative timing, minimum timing of maximum distance between screener-screener-defender and minimum of user-user-defender and screener-user-defender was examined.

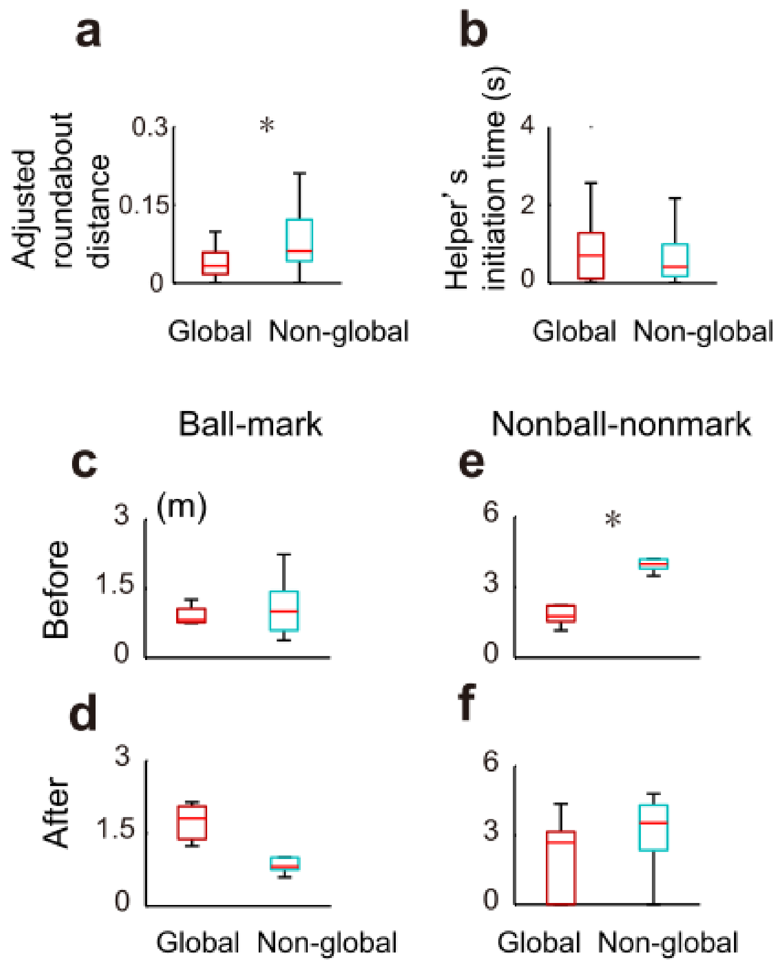

Figure S14. Global and non-global helping behaviour. (a) Adjusted roundabout distance between global and non-global helping behaviour. (b) Helper's initiation time relative to the start of screen. (c-f) Adjusted spatial-gap at 2 subsystem scales remaining in Fig. 6 of 5-on-5 system before (c,e) and after (d,f) the screen.

3. Supplementary tables

Table 1. Threshold in detecting plays.

**Parameter**	**Threshold**
Horizontal distance from attacker during ball holding	1 m
Horizontal acceleration during ball catching	–5 m/s^2
Vertical position of ball during shooting	3.2 m<br>(ring height: 3.05 m)
Horizontal distance between ball and ring during shooting	0.5 m<br>(ring diameter: 0.45 m)
Distance of switching marking attackers	2 m
Distances of approaching detecting screen between screener and user-defender and both attackers	1 m
Desired defensive distance from attacker's position	0.5 m
Maximum roundabout distance during screen	1 m
Minimum peak acceleration to detect initiation	3 m/s^2
Minimum peak acceleration interval for initiation	0.1 s
Minimum velocity for initiation	1 m/s
Approaching distance to detect various plays	3 m

Table 2. Results of logistic analysis in successful shot.

Category	Distance	Nagelkerke R^2	Hosmer-Lemeshow test (p)	standard partial regress. coeff. (B)	Wald test (p)	Odds ratio	95% lower confidence bound	95% upper confidence bound	AIC
Entire period	Ball-mark (both)	0.17	0.98	1.85	0.012	6.38	1.52	26.88	81.1
(Fig.2b,d,	Ball-nonmark (both)	0.13	0.19	1.23	0.019	3.43	1.22	9.63	83.1
n = 61)	Nonball-mark (both)	0.12	0.12	1.09	0.029	2.98	1.12	7.91	83.7
	Ball-mark (spatial)	0.14	0.74	1.32	0.019	3.73	1.25	11.18	82.4
Before shot	Ball-mark (both)	0.18	0.87	1.95	0.009	7.01	1.62	30.31	80.4
(Fig.S9b,d,	Ball-nonmark (both)	0.09	0.43	1.34	0.049	3.83	1.01	14.59	84.8
n = 61)	Ball-mark (spatial)	0.14	0.82	1.29	0.015	3.64	1.29	10.31	82.5
	Ball-nonmark(spatial)	0.12	0.14	1.09	0.029	2.98	1.12	7.91	83.7
After screen (Fig.S2, n = 41)	Ball-mark (both)	0.26	0.28	1.68	0.010	5.34	1.50	19.05	53.5

4. Legends of supplementary videos

Movie 1. Attacker-defender distance adjusted in heterogeneous environment. Time series are the same as Fig. S1a.

Movie 2. Adjusted attacker-defender distance at 4 subsystem scales. Time series are the same as Fig. S1b.

Movie 3. Screen to break person-to-person rule. Attacker 5 (screener) executed a screen for attacker 3 (user) to break the defensive person-to-person rule and attacker 3 made a shot.

Movie 4. Leaving the screen. Defender 5 left the screen (by attacker 5) and defender 5 and 3 kept the defensive person-to-person rule.

Movie 5. Transient help during screen. Defender 4 transiently helped defender 1 and returned the original role.

Movie 6. Role-switching of defenders during screen. Defender 3 and defender 5 (triangles) switched their roles.

Movie 7. Global help during screen. Defender 4 ignored the screen involved with him and helped another teammate.
